# Black Thyroid Associated with Thyroid Carcinoma

**DOI:** 10.1155/2010/681647

**Published:** 2010-11-23

**Authors:** Emad Kandil, Mohamed Abdel Khalek, Haytham Alabbas, Philip Daroca, Tina Thethi, Paul Friedlander, Ryan Leblanc, Obai Abdullah, Bernard Jaffe, Byron Crawford

**Affiliations:** ^1^Division of Endocrine and Oncologic Surgery, Department of Surgery, Tulane University School of Medicine, New Orleans, LA 70112, USA; ^2^Department of Pathology, Tulane University School of Medicine, New Orleans, LA 70112, USA; ^3^Department of Physiology, Tulane University School of Medicine, New Orleans, LA 70112, USA; ^4^Division of Endocrinology, Department of Medicine, Tulane University School of Medicine, New Orleans, LA 70112, USA

## Abstract

*Objective*. Black thyroid is a rare pigmented change seen almost exclusively in patients upon minocycline ingestion, and the process has previously been thought to be generally benign. There have been 61 reported cases of black thyroid. We are aware of 13 cases previously reported in association with thyroid carcinoma. This paper reports six patients with black thyroid pigmentation in association with thyroid carcinoma. *Design*. The medical records of six patients who were diagnosed with black thyroid syndrome, all of whom underwent thyroid surgery, were reviewed. Data on age, gender, race, preoperative fine needle aspiration biopsy (FNA), thyroid function levels, and pathology reports were collected. *Main Outcome*. The mean age was 60 years. There were 5 females, 4 of whom were African American. All patients were clinically and biochemically euthyroid. Black pigmentation was not diagnosed in preoperative FNA, and only one patient had a preoperative diagnosis of papillary thyroid carcinoma. The other patients underwent surgery and were found to have black pigmentation of the thyroid associated with carcinoma. *Conclusions*. FNA does not diagnose black thyroid, which is associated with thyroid carcinoma. Thyroid glands with black pigmentation deserve thorough pathologic examination, including several sections of each specimen.

## 1. Introduction

Black thyroid is a rare condition. A recent review by Oertel and colleagues has identified 61 cases that have been reported to date [1]. Since the first report of black thyroid in human tissue [2], the etiology has been associated with minocycline ingestion for year or more [2, 3]. Although minocycline has been suggested as the cause of the pigmentation in many cases [4–6], it has also been noted to occur subsequent to infection [7], perhaps due to treatment with other tetracycline derivatives [3].

Prescribed as an antibiotic for acne vulgaris, minocycline has shown a rare yet seemingly specific relationship with black thyroid [8]. The drug reacts with thyroid peroxidase and forms a black pigment which could be readily seen in histological preparations [6, 9–11]. It has been recommended that (because of its antioxidant properties) coadministration of ascorbic acid can protect against the discoloring effects of minocycline [12]. 

Often presenting with hypothyroidism, hyperthyroidism, neck swelling, or no symptoms [8, 13–15], black thyroid is distinguished by its melanin-like pigmentation and a lack of autoimmune fluorescence, iron deposits, or lipofuscin while bleaching with potassium permanganate [7]. 

It has been recently recognized that black thyroid may be involved in thyroid carcinoma, as the reported comorbidity approximates 40% [16]. Thus, the recognition of black thyroid necessitates serious consideration of coincidental malignancy.

 

## 2. Materials and Methods

The medical records of six patients who were diagnosed with black thyroid syndrome between January 2005 and December 2007, all of whom underwent thyroid surgery, were reviewed. Data on age, gender, race, preoperative fine needle aspiration biopsy (FNA), thyroid function levels, and pathology reports were collected (Table 1). 

## 3. Results

**Figure 1:**
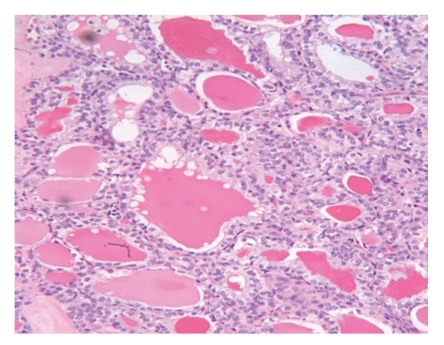
Follicular variant of papillary thyroid carcinoma.

**Figure 2:**
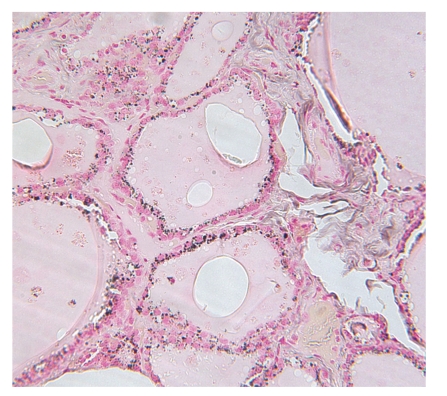
Fontana-Masson stain reduces ammonia-silver nitrate and turns black (positive) in black thyroid.

The mean age was 60 years (±5.9). There were 5 females, and 4 of whom were African Americans. All patients were clinically and biochemically euthyroid. Black pigmentation was never diagnosed in preoperative FNA, and only one patient had a preoperative diagnosis of papillary thyroid carcinoma (Figure 1). Other patients underwent surgery for compressive symptoms, suspicious nodules on ultrasound (nodule size >1 cm), or family history of thyroid cancer. All patients were found to have black pigmentation (Figure 2) of the thyroid. All patients were found to have thyroid carcinoma on postoperative pathological examination (Table 1).

## 4. Discussion

Thyroid cancer occurs more commonly in women [17], with papillary cancer being the most common type of thyroid cancer. According to Mitchell and associates, the northeastern and southern areas of the US have the highest incidence [17]. The current series demonstrates that the high incidence of black thyroid in this region of the country is consistent.

The most relevant side effects of minocycline are dizziness, nausea, diarrhoea, hyperpigmentation of the skin, and a macroscopic black discoloration of the thyroid gland, designated ‘black thyroid syndrome'. This black discoloration of the thyroid is almost pathognomonic for the use of minocycline [8]. Although many case reports describe an association between minocycline-induced black thyroid and malignancy [13, 16–19], a causal relationship has never been proven. Hecht et al. described seven cases with black thyroid with no malignancies [13].

Birkedal and coworkers have recommended that incidental discovery of black thyroid should be dealt with proactively with surgical resection to stem the risk of papillary thyroid carcinoma [16]. To further this contention, a literature review in 2005 by Bruins and colleagues reported a 30% (9/30) incidence of carcinoma in patients presenting with black thyroid as opposed to a 0.003% (3/100,000) incidence in the general population [8]. Although the difficulty of diagnosis with fine needle aspiration has been noted [1, 20], past minocycline use significantly increases the suspicion of black thyroid, raising the question as to whether more definitive measures are warranted to prevent carcinoma in situ.

## 5. Conclusion

Thyroid glands with black pigmentation deserve thorough pathologic examination, which should include evaluation of increased numbers of blocks sampled from each specimen. However, these findings warrant further investigation to clarify the exact incidence of thyroid carcinoma in association with black thyroid pigmentation.

##  Conflict of Interests

No competing financial interests exist for all authors.

## Figures and Tables

**Table 1:** Patients' demographics and pathology information.

	Patient 1	Patient 2	Patient 3	Patient 4	Patient 5	Patient 6
Age	68	52	57	59	58	66
Gender	Female	Male	Male	Female	Female	Female
Race	White	African American	White	African American	African American	African American
Preop TSH(reference range: 0.5 and 3.0 mIU/L)	1.84	1.47	1.33	1.52	1.24	1.38
Preop FNA	Benign nodule	Benign nodule	Follicular lesion	Benign nodule	Papillary thyroid cancer	Follicular lesion
Pathology	Papillary thyroid cancer	Hurthle cell neoplasm	Follicular carcinoma	Hurthle Cell Neoplasm	Papillary thyroid cancer	Papillary thyroid cancer
